# Study of Self-Heating and High-Power Microwave Effects for Enhancement-Mode p-Gate GaN HEMT

**DOI:** 10.3390/mi13010106

**Published:** 2022-01-09

**Authors:** Yingshuo Qin, Changchun Chai, Fuxing Li, Qishuai Liang, Han Wu, Yintang Yang

**Affiliations:** Key Laboratory of Ministry of Education for Wide Band-Gap Semiconductor Materials and Devices, School of Microelectronics, Xidian University, Xi’an 710071, China; ysqin_chin@163.com (Y.Q.); ccchai@mail.xidian.edu.cn (C.C.); liangqishuai428@163.com (Q.L.); 15686041593@163.com (H.W.); ytyang@xidian.edu.cn (Y.Y.)

**Keywords:** GaN, self-heating effect, high-power microwave, thermal mechanism, HEMT, damage area prediction

## Abstract

The self-heating and high-power microwave (HPM) effects that can cause device heating are serious reliability issues for gallium nitride (GaN) high-electron-mobility transistors (HEMT), but the specific mechanisms are disparate. The different impacts of the two effects on enhancement-mode p-gate AlGaN/GaN HEMT are first investigated in this paper by simulation and experimental verification. The simulation models are calibrated with previously reported work in electrical characteristics. By simulation, the distributions of lattice temperature, energy band, current density, electric field strength, and carrier mobility within the device are plotted to facilitate understanding of the two distinguishing mechanisms. The results show that the upward trend in temperature, the distribution of hot spots, and the thermal mechanism are the main distinctions. The effect of HPM leads to breakdown and unrecoverable thermal damage in the source and drain areas below the gate, while self-heating can only cause heat accumulation in the drain area. This is an important reference for future research on HEMT damage location prediction technology and reliability enhancement.

## 1. Introduction

After decades of evolution of semiconductor process technology, the semiconductor process has already entered the nano era, and the heat accumulation problem worsened by higher integration has become more and more serious. The wide bandgap semiconductor device gallium nitride high electron mobility transistor (GaN HEMT), with its advantages of high critical breakdown electric field, high temperature tolerance, high output power density, and high efficiency [1,2,3,4,5], is used in 5G, satellite communications, radar, radio frequency, aerospace, and other fields. GaN-based devices can make up for the deficiencies of silicon-based devices due to the limitations of the traditional semiconductor materials, becoming one of the most promising candidates in the high-frequency and high-power area [4,6,7]. High output power means greater thermal generate power, and the resulting chip performance degradation is also an important reason for the large gap between actual working efficiency and theoretical efficiency [8,9,10], and so the heating problem needs to be taken seriously.

Semiconductor devices generate heat during normal operation due to self-heating, making the temperature of critical areas much higher than the basic ambient temperature. This not only affects the reliability, but it is also a key factor in reducing the lifespan of the equipment [11]. High-power microwaves, as a kind of strong electromagnetic pulse (EMP), can be coupled into the electronic system through antennas, sensors, and gaps in the system package shell, etc., causing the electronic system to generate large noise signals and malfunctioning. At worst, it can result in equipment failure and unrecoverable thermal damage [12]. In addition, with the reduction in the size of electronic equipment, working voltage, and power consumption, and the improvement of system integration, electromagnetic systems are becoming more and more sensitive to the external environment; the damage threshold is lower and lower, and the problem of heat accumulation is becoming more and more serious. Therefore, the study of these two thermal effects has very important practical significance for the improvement of the robust performance of future electronic equipment systems.

The vulnerability of electronic system failures has increased in recent decades due to intentional electromagnetic interference (IEMI). One of the reasons is that electronic systems are becoming more and more sensitive to the external environment due to the increase in integration. The rapid development of electromagnetic interference generation technology is another factor that cannot be ignored [13]. At the beginning of the Gulf War in 1991, a stealth “F-117” aircraft with an “experimental tactical microwave missile warhead” appeared over Baghdad. After the explosion, a large number of Iraqi military electronic equipment near the blast site suddenly failed [14]. In the 2003 Iraq War, U.S. forces used electromagnetic pulse bombs to paralyze the television broadcasting system of the Iraq National Television for a period of time [15]. The two attack cases have one thing in common, that is, the overall electronic equipment did not appear to be damaged, but some of the electronic devices inside of the equipment were mysteriously destroyed. In September 2001, Rosoboron Export Company of Russia exhibited a mobile microwave radio frequency (RF) beaming system named “Ranets-E”, which can reduce the accuracy of weapons within a distance of 10 km. The output power is above 500 MW, and the transmission pulse width is 10–20 ns [14].

At present, the study of the self-heating effect mainly focuses on the degradation of performance and ways to weak this negative effect. X. Wang et al. studied the negative differential conductance (NDC) phenomenon caused by self-heating, and gave a new structure that suppresses the effect [16]. Chattopadhyay et al. developed a charge control model for thermal analysis of GaN HEMT and found that the increase in temperature will decrease mobility [17]. A. Jarndal et al. developed a large-signal model of GaN HMET, which takes into account the performance degradation caused by temperature rise and can accurately predict self-heating-induced current dispersion [18]. T. Zhu et al. plated a diamond layer above the passivation layer for GaN HEMT, which can improve heat dissipation [19]. Due to its military value, the study of HPM effects has also become a hot spot in recent decades. Y. M. Zhang et al. studied the thermal characteristics of the GaN HEMT device under the electrical pulse of different duty cycles and frequency and found that low duty cycle and high-frequency operation are beneficial in improving lifetime and performance reliability [20,21]. Y. Q. Liu et al. found that HPM can cause the non-linear response of HEMT, and the research results show that the cause of this phenomenon is tunneling and impact ionization [8]. Q. S. Liang et al. studied the impact of HPM radiation on CMOS and established a numerical model that can calculate the internal dominant current [22]. L. Zhou et al. applied HPM pulses to the GaN HEMT to study the damage of thermal stress to the device, and the results showed that the field plate near the gate is a vulnerable area [23]. However, these two thermal effects have different mechanisms and effects on semiconductor devices, which were not mentioned in the previously reported work.

In this paper, an enhancement-mode p-gate GaN HEMT model is established. The enhancement-mode (E-mode) GaN HEMTs possess inherent advantages including low power consumption, enhanced system safety, better frequency characteristics, and simple circuit configuration compared with the depletion-mode (D-mode) devices [24]. The distributions of lattice temperature, energy band, current density, electric field strength, and carrier mobility within the device are plotted to facilitate analyzing the mechanical behavior of the two effects from a physical point of view and compare their differences. The simulation results are verified by previously reported work and provide an important reference for the future research of HEMT device damage location prediction and reliability reinforcement.

## 2. Device Structure and Simulation Model

**Figure 1:**
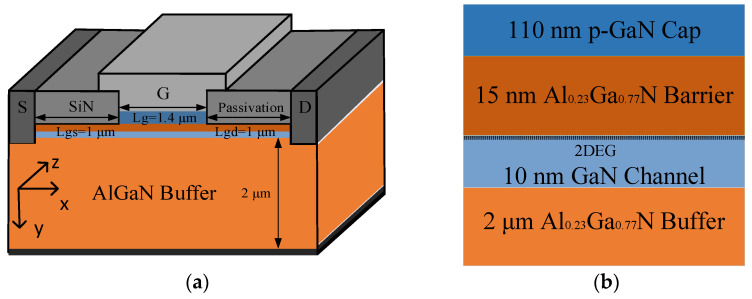
(**a**) Cross-sectional schematic of the enhancement-mode p-gate AlGaN/GaN HEMT; (**b**) partially enlarged view under the gate.

A two-dimensional device model is built in this paper. In the specific simulation process, Sentaurus TCAD will assume a “thickness” (effective width along the z-axis) of 1μm. The cross-sectional schematic of the enhancement-mode AlGaN/GaN heterostructure HEMT is shown in Figure 1a. The physical models of AlGaN/GaN HEMT adopted in this simulation consist of a 10 nm thick AlN nucleation layer,2 μm thick Al_0.05_Ga_0.95_N buffer layer, a 10 nm thick GaN channel layer, a 15 nm thick Al_0.23_Ga_0.77_N barrier layer, a 0.2 μm thick SiN passivation layer, and a 110 nm thick p-GaN cap layer. The length of the gate Lg, the gate-source extension Lgs and the gate-drain extension Lgd are 1.4 μm, 1 μm, and 1 μm, respectively. The device is 4.4 μm wide. The doping concentrations of the p-GaN layer, AlGaN barrier layer, GaN channel layer, and AlGaN buffer layer are 3 × 10^17^ cm^−3^, 1 × 10^18^ cm^−3^, 1 × 10^15^ cm^−3^, and 1 × 10^14^ cm^−3^, respectively.

A part of oxygen atoms or nitrogen atoms will be generated which makes AlGaN buffer show slight N-doping characteristics due to the AlGaN epitaxial growth process [25]. In this paper, a light N-type doping is applied to simulate this effect. The SiN passivation layer can moderate the current collapse effect to a certain extent [26]. P-GaN cap layer is used to elevate AlGaN/GaN heterojunction conduction band that makes an enhancement-mode device [27]. The channel layer is doped to increase the two-dimensional electron gas (2DEG) electrons concentration. The 2DEG electrons are mainly from Al_0.23_Ga_0.77_N barrier layer doping. The concentration of 2DEG electrons will be slightly affected by the polarization effect and surface state as well [28].

The simulation work of this study was performed by Sentaurus TCAD software [29]. Some material parameters used in this work such as mobility *µ*, bandgap *Eg*, and thermal parameters are related to temperature(*T*) or other factors. Take mobility as an example, it is constrained by factors such as electric field, doping concentration, and temperature. The carrier mobility at different moments and positions is different. The initial values of some parameters are shown in Table 1. However, using initial values during the entire simulation process will lead to an inaccurate simulation result, so it is important to consider necessary physical models to calibrate the simulation. Default values are used for other material parameters in Sentaurus TCAD. A brief description is given below:

The Fermi statistical distribution model is applied for heavily doped active regions to characterize that when the doping concentration exceeds 1 × 10^19^ cm^−3^, Boltzmann statistics can no longer be used to approximate Fermi statistics. The Fermi model should be chosen to improve simulation accuracy.

With the effect of scattering, the constant mobility model that previously reported work used cannot match the actual movement of carriers well. Dynamic mobility model was applied which includes lattice scattering effect, ionized charge scattering effect, carrier scattering effect, doping-dependence effect, and high-field-saturation effect.

Piezoelectric polarization model is involved for the polarization effect that is unique to GaAs HEMTs. The electric field induced by the spontaneous and piezoelectric polarization can result in an increase in the carrier concentration at the AlGaN/GaN heterointerface [28]. In our work, the interface charge induced by the polarization at AlGaN/GaN is set as 6 × 10^12^ cm^−2^. Sentaurus provides two models (strain and stress) to compute polarization effects in GaN devices. The effect of piezoelectric polarization is simulated by adding the divergence of the piezoelectric polarization vector as an additional charge term. Traps can be coupled to nearby interfaces and contacts through tunnels. Sentaurus models non-local tunneling to the trap as the sum of inelastic, phonon-assisted process and elastic process [30,31]. The Barrier-tunneling model and traps model are activated. The interface defect traps density of AlGaN/Nitride(donor-type), AlGaN/Oxide(acceptor-type), and GaN/AlGaN (acceptor-type) were set as 5 × 10^13^ cm^−2^, 5 × 10^13^ cm^−2^, and 6 × 10^12^ cm^−2^, respectively. In addition, that of the buffer region (acceptor-type) is 1 × 10^15^ cm^−2^.

The thermionic model is activated since the conventional transport equation is no longer valid at the heterojunction interface, and the current and energy flux at the abrupt interface between the two materials were used to define the interface conditions of the heterojunction [32]. The Shockley Read Hall recombination model and recombination-generation-heat model are activated to calibrate the recombination effects. Besides, the thermodynamic is activated to simulate the electrothermal characteristics by extending the drift diffusion under the assumption that carriers are in thermal equilibrium with the lattice. The change of lattice temperature and current density was affected by the above thermal models sensitively. The boundary of the device was set as the thermal contact surface and the substrate bottom was set to be 300 K.

## 3. Results and Discussion

Based on the above physical and the geometrical models, simulations were carried out from the dimensions of electricity and thermal conductivity, and a series of results were obtained for analysis and discussion which fit with the experiments well.

### 3.1. Electrical Characteristics

**Figure 2:**
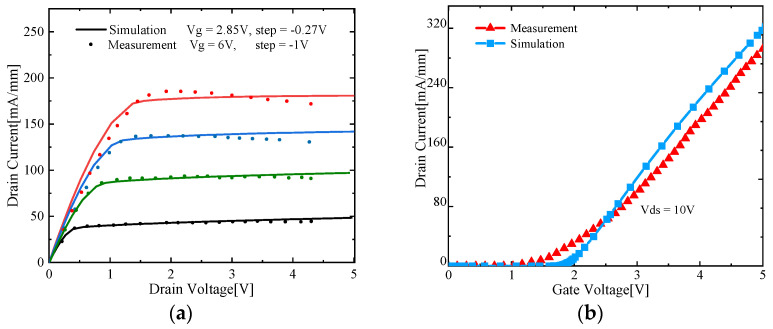
(**a**) Simulation and measurement comparison in output characteristic (simulation—solid line; measurement—dotted line); (**b**) transfer characteristic (simulation, blue line; measurement, red line).

Figure 2 shows the simulation results which fit the previously reported experimental data in appearance [33]. The transfer characteristics curve and output characteristics curve agree with the experimental measurement well. The value deviation between simulation and experiment in output current density, threshold voltage, and turning point of the I_D_-V_D_ curve is caused by the structure and material properties. The proportion of aluminum in the AlGaN layer, the doping concentration in the barrier layer, and the geometric size of the device that the simulation adopted all contribute to the slight deviation [33,34]. The above results demonstrate the reliability of the simulation model in this paper.

**Figure 3:**
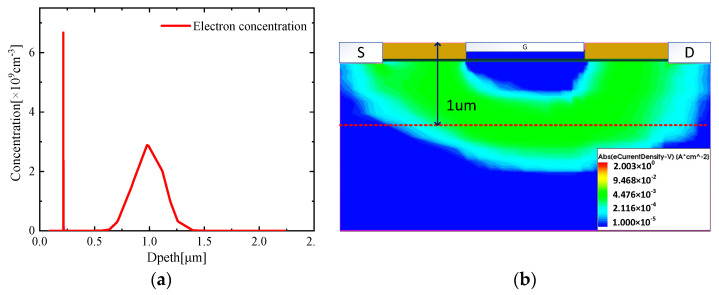
(**a**) Off-state carrier density distribution diagram; (**b**) current density distribution of the device when Vg = 0 V, Vds = 18 V.

**Figure 4:**
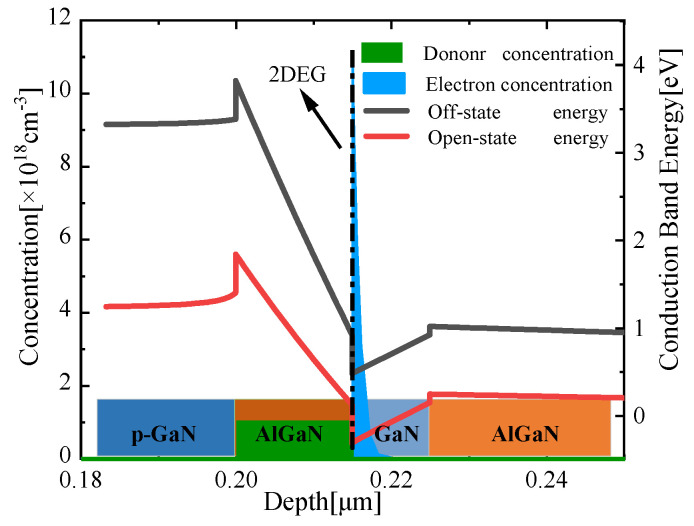
Simulated band diagram (red and grey line, right Y-axis) of the HEMT and electron distribution (green and blue zone, left Y-axis) among different layers.

For HEMT semiconductors, it is far from enough to only study its IV characteristics; its internal mechanism also needs to be revealed. Before being turned on, there is a certain concentration of carriers in the channel layer as shown in Figure 3a, which cannot provide the normal work mode. What cannot be ignored is a carrier distribution of an order of magnitude of a normal distribution pattern under the channel layer. The off-state studied here refers to the situation that the gate voltage is less than the threshold voltage, and the voltage bias of the source and drain electrodes is normal (Vds = 18 V). The carriers of the barrier layer, the channel layer, and the buffer layer are led to both ends under the action of the source–drain potential difference, forming a weak leakage current path in the buffer area, which is obvious from the current density distribution results as shown in Figure 3b. After the channel is turned on, most of the carriers are concentrated in the two-dimensional electron gas (2DEG) layer. The carrier concentration under the channel is negligible compared with the carrier concentration in the 2DEG layer. The difference in electron distribution between on-state and off-state can be explained from the conduction band diagram in Figure 4: before the HEMT is turned on, there is a potential barrier of up to 3.8 eV, and only a small part of the carriers can pass through the barrier and enter the channel layer. As the channel turns on, the conduction band moves down to 1.8 eV due to the external gate voltage, and most of the carriers in the AlGaN barrier layer can enter the channel layer and be trapped in the quantum well to form a two-dimensional electron gas. Most of the carriers are concentrated in the direction perpendicular to the AlGaN/GaN interface which is defined as 2DEG.

**Figure 5:**
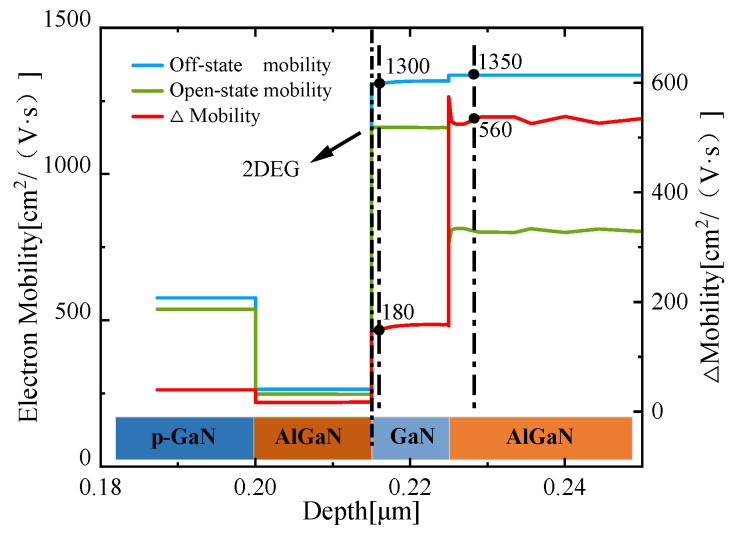
The electron mobility (blue and green line, left Y-axis) and ∆mobility (red line, right Y-axis) in the enhancement-mode p-Gate AlGaN/GaN HEMT of different operation modes.

With the heterojunction structure, the HEMT device separates the ionization center and free electrons spatially which aims to gain higher mobility for carriers. At the initial off-state moment, the lower carrier concentration in the channel layer leads to a higher mobility due to the weak scattering effect. With the injection of the gate voltage, the channel is turned on, the concentration of the two-dimensional electron gas rises sharply, and the scattering between the carriers is enhanced. With the accumulation of heat, the temperature rise leads to the enhancement of the irregular thermal movement of the carriers and the scattering is further improved. Under the influence of the above-mentioned negative effects, the carrier mobility of the two-dimensional electron gas layer, AlGaN buffer layer, has dropped by 13.85% and 41.48%, respectively, as shown in Figure 5. The difference can be explained as follows: the carriers in the buffer layer are scattered in the horizontal and vertical directions, while the carriers in the 2DEG layer are only scattered in the horizontal direction.

**Figure 6:**
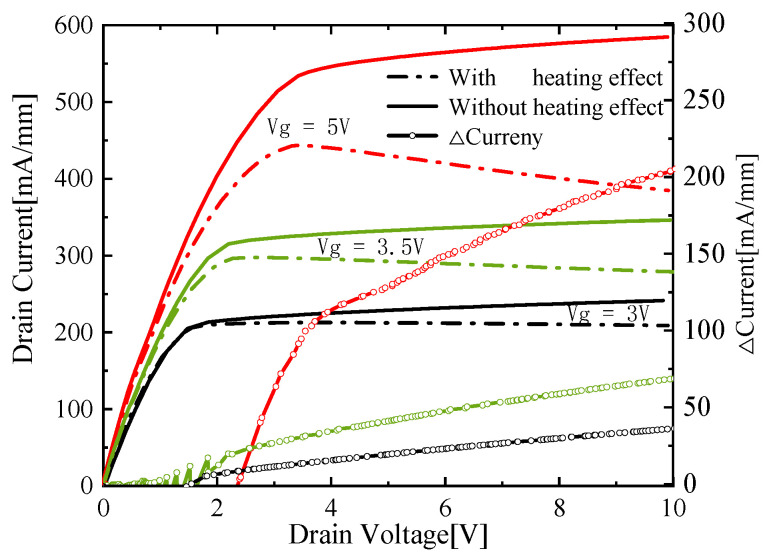
Diagram of drain current with drain voltage for various values of gate voltage with self-heating (solid line, left Y-axis), without self-heating (dotted line, left Y-axis), and ∆current (solid line with hollow circle, right Y-axis).

It can be seen from Figure 6 that the decrease in mobility results in a drop of current which leads to an evident negative differential conductance (NDC) phenomenon [35]. When the device is just turned on, the current has no obvious downward trend. With the continuous increase in the inject voltage, the current decreasing trend caused by the temperature rise effect increases due to the negative effects discussed above. Besides, the current decrease caused by the temperature rising effect could neutralize the current increase by the channel length modulation effect in output characteristics. The channel length modulation effect is further weakened with the increase in gate voltage that makes the overall output characteristics no longer show the features of the channel length modulation effect. This trend aggravates as the drain voltage enlarges.

### 3.2. Thermal Characteristics

Joule heat is generated by the interaction of electric current and resistance. Semiconductor devices will generate heat during normal operation, especially HEMTs composed of third-generation wide-gap semiconductor material with the high output power density and high-frequency characteristics. The increase in temperature will cause performance degradation and even unrecoverable damage to the device [8,19,36], which contributes to an obvious gap between actual performance and theoretical performance of HEMTs. Therefore, in order to improve the working efficiency of HEMTs, the temperature rise phenomenon needs to be studied emphatically.

#### 3.2.1. Self-Heating Effect

**Figure 7:**
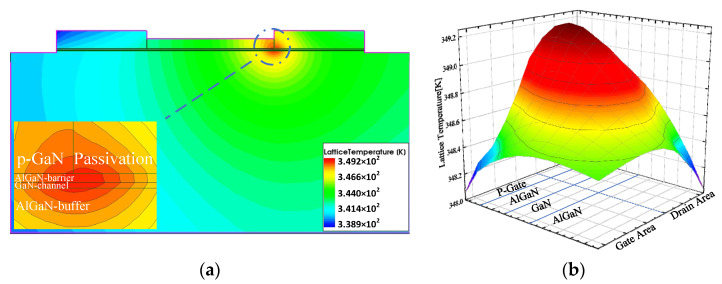
(**a**) Internal thermal distribution of the device under self-heating effect; (**b**) thermal distribution near the hot spot.

**Figure 8:**
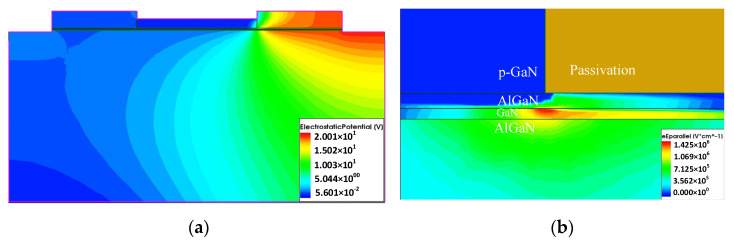
(**a**) Internal electric potential distribution of the device with self-heating effect; (**b**) electric field distribution near the hot spot.

Figure 7a depicts the thermal distribution of the device. Specifically, the hot spot is located in the channel region near the junction of the gate and the passivation layer. Figure 8a shows that the hot spot is the area with the largest potential gradient, where the electric field strength is also the greatest. This can be explained well in theory due to the output simulation bias conditions. When performing output characteristic simulation, the source voltage is set to 0 V, the gate voltage is set to 2 V, and the drain voltage is scanned from 0 V to 18 V. The voltage drop between the gate and the drain is much bigger than the voltage drop between the gate and the source. The electric field is proportional to the voltage across the two electrodes due to the same gate-source distance Lgs and gate-drain distance Lgd. Thus, the maximum electric field strength is located the drain area, which is consistent with the distribution result of Figure 8b.

**Figure 9:**
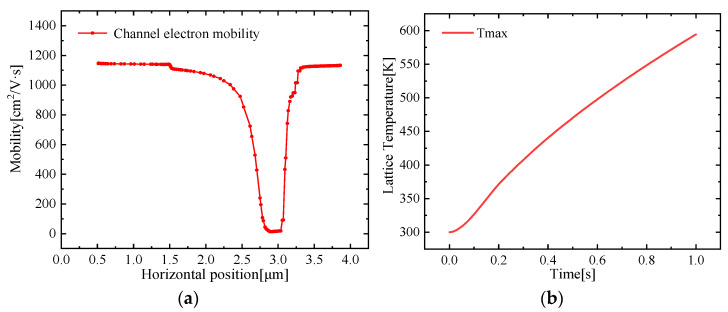
(**a**) Variation of electron mobility in the horizontal direction of the channel layer; (**b**) *Tmax* curve with self-heating effect over time.

The action of a strong field leads to a larger current density, resulting in more Joule heat. An increase in temperature leads to a decrease in mobility, as shown in Figure 9a, which is a very good explanation for the NDC phenomenon mentioned above. In addition, the resistivity will also increase with the temperature, the current will decrease, and the rate of temperature rise will slow down which can be clearly demonstrated in the HPM effect discussed below. Figure 9b shows self-heating temperature variation over time; it is found that the temperature rise rate increases with time in the initial stage under self-heating conditions. At a certain time node, the temperature increases almost at a relatively constant rate.

#### 3.2.2. HPM Effect

HPM radiation is typically defined as comprising a frequency content between 300 MHz and 300 GHz, with a peak power of not less than 100 MW but often reaching the gigawatt level [37]. Large-scale military-grade electronic attack systems can generate peak electric fields of hundreds of kilovolts per meter to permanently destroy most unshielded electronic devices [38]. In the laboratory, HPM generation is performed by a pulse machine (PPM), which can generate voltages up to several thousand volts for 100 ns [39]. The peak power of the electromagnetic signal generated by the HPM source can reach hundreds of watts or even gigawatts, which can be coupled to the electrode ports of critical devices in the form of hundreds of volts. Compared with the irradiation method, the injection method can better amplify the damage degree of HEMT and reduce the damage time threshold when HPM effect is investigated. In the numerical simulation, HPM does not act on the circuit port in the form of an electromagnetic field, but the equivalent voltage or current signal. Previously reported work concludes that the narrow-band HPM coupling voltage can be characterized by an unattenuated sinusoidal voltage signal [40]. In this work, a sine wave as the specific HPM signal with constant power (300 V, 5 GHz) is injected into the gate. Vds is biased as 1 V and defines the melting point of GaN (1973 K) as the burn-out threshold.

**Figure 10:**
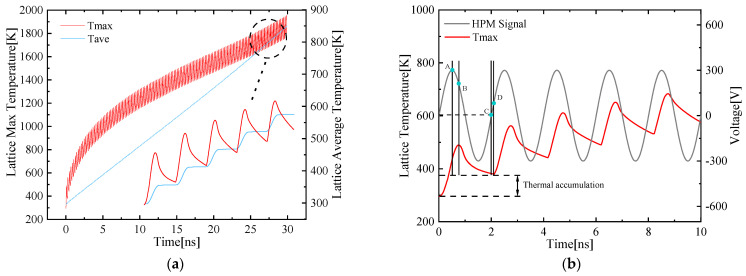
(**a**) Variation of *Tmax* (maximum temperature, red line, left Y-axis) and *Tave* (average temperature, blue line, right Y-axis) over time inside the device; (**b**) *Tmax* (red line, left Y-axis) and HPM signal (grey line, right Y-axis) change trend within 10 ns.

It can be seen from Figure 10a that the maximum temperature (Tmax, red line) inside the device rises rapidly in the initial stage, and gradually slows down over time while the average temperature (Tave, blue line) is increased at a relatively stable rate. In a temperature rise period, the change of *Tmax* is more obvious than *Tave* as shown in Figure 10b. This can be understood as the accumulated thermal is averaged to the entire device, and the temperature rise is relatively slow.

It can be seen from the *Tmax* line in Figure 10a that the temperature rises steeply during the initial time period and then tends to stabilize. The specific waveform of *T* is similar to a sinusoidal signal with a rising trend that presents a step-like increase, and there is a slight hysteresis between the HPM signal and *Tmax* waveform as shown in Figure 10b. On the one hand, because the accumulation of thermal energy takes a certain amount of time, the temperature change is continuous as the heat transfer takes time while the voltage does not. Another detailed explanation is that the dissipated power is related to the device temperature. The thermal accumulation leads to a larger temperature gradient between the device and the external environment which results in a greater dissipated power.

Take the first temperature rise period as an example: at the moment the HPM signal is injected, the temperature rises with the accumulation of heat. The voltage amplitude corresponding to this initial temperature rise point is defined as the threshold value *Vt*. The temperature keeps rising when the HPM signal reaches the peak of the first clock cycle (point A), indicating that the heat accumulation rate at this time is still greater than the heat dissipation rate. After the next 1/4 clock cycle, the HEMT device temperature reaches the first peak value (point B). At this moment, the dissipated power also reaches maximum because the temperature gradient between the device and the external reaches the maximum during this period. The two realize a dynamic balance, the rate at which the temperature rises ∆T/∆t = 0. With additional time, the device temperature keeps dropping due to the net difference between the cumulative power of the heat and the external dissipation power.

The temperature of the device at the end of the first temperature cycle (point C) is higher than the initial time, indicating that the heat has achieved a net accumulation with the action of the HPM signal. From the end of the first clock cycle to the beginning of the second clock cycle, the device temperature keeps dropping until the HPM signal reaches a certain amplitude (point D), that is, the second threshold temperature rise point, the heat can achieve a net accumulation.

**Figure 11:**
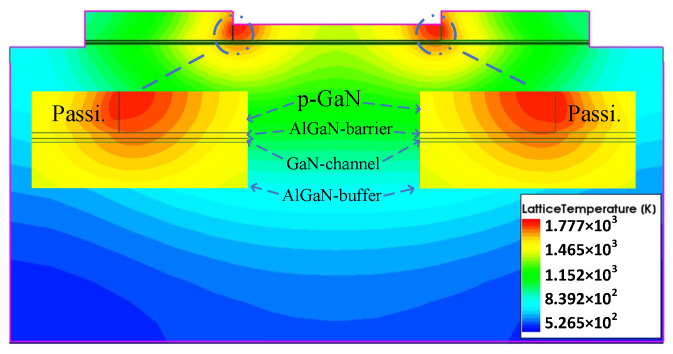
The internal thermal distribution of the device under HPM effect.

Figure 11 shows the thermal distribution under HPM injection which behaves slightly different from that in self-heating mode. It can be seen that the center of the thermal source is located on both sides of the gate close to the source and drain.

**Figure 12:**
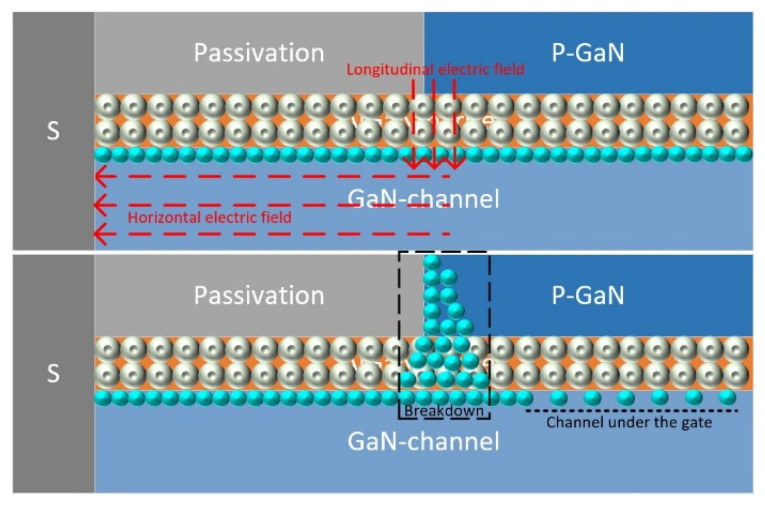
Thermal damage mechanism description of the hot spot.

Taking the source as an example to analyze the detailed damage process due to the symmetry, Figure 12 depicts the distribution of electrons near the damage zone. With the injection of the HPM signal, there is a huge horizontal potential difference between the gate and side electrode which results in a region of maximum electric field strength. In the vertical direction, the free electrons in the barrier layer enter the quantum well of the two-dimensional electron gas layer, leaving a large amount of ionized central charges. The very thin barrier layer also promotes the formation of maximum field strength. The intersection of the maximum of the horizontal field strength and the maximum of the vertical field strength constructs a potential breakdown zone. When the breakdown occurs, a large number of electrons pass directly through the p-GaN side zone which contributes a great current. Joule heat is generated sharply in the area where the breakdown current flows.

**Figure 13:**
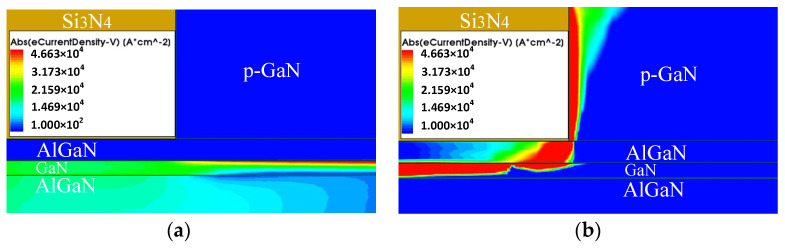
(**a**) Electron current distribution near the source before breakdown; (**b**) electron current distribution near the source after breakdown.

Figure 13 shows the electron current distribution near the source at the different moments extracted from the simulation software which fit the above hypothesis well. Before the breakdown, electrons are concentrated in the two-dimensional electron gas layer, where the temperature gradually rises. With the accumulation of heat, the internal energy of electrons and atoms continues to increase, and the probability of breakdown occurring increases with time, and finally reaches the breakdown threshold, forming a large breakdown current across the channel, AlGaN-barrier, and p-GaN region. As shown in Figure 13b, the breakdown zone shares most of the current in the channel region under the gate and makes the channel almost off. The reaction of the breakdown current and resistance in this area generates a large amount of Joule heat, which causes the temperature of the HEMT device to increase until it burns out.

**Figure 14:**
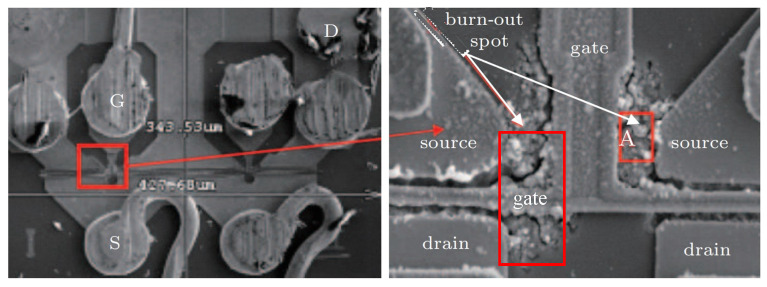
Interior features of damaged samples, characterized by SEM.

To verify the above explanation further, the experiment was performed by directly injecting high-power microwaves into a low-noise amplifier (LNA) containing a pHEMT device [36]. The injected HPM signal is a continuous wave with high-density power, generated by a synthesized sweeper (HP 83630 A) modulated by a pulse generator (HP 81130 A). In the experiment, it is difficult to extract the internal temperature distribution of the device, and the LNA noise figure deterioration of 20 dB is defined as the damage threshold [41]. We observed the internal features with a scanning electron microscope. From the results of the scanning electron microscope, as shown in Figure 14, it can be seen that obvious burnout occurred in the area of the gate close to the source and drain. There are multiple melting pits in this area, which are caused by the high current density. Breakdown makes the device partially melt, and the damage position is verified by the previous analysis of this paper. It is also consistent with the experimental results discussed in the literature [42].

## 4. Conclusions

In this paper, an enhancement-mode p-gate AlGaN/GaN HEMT consistent with the experimental results is established, and the self-heating and high-power microwave effects are investigated from electric and thermal aspects.

The research results of the self-heating effect show that there is an obvious variation in energy band and carrier distribution when the channel is turned on. Coupled with the influence of various scatterings and temperature rise, the electron mobility decreases which results in the attenuation of output current. In addition, the area where the hot spot under the self-heating effect is located, the area with the largest electric potential gradient, and the area with the largest electric field strength are highly coincident.

In the simulation of the HPM effect, the thermal behavior of the device was discussed and the mechanism of the hysteresis phenomenon was explained from the perspective of dynamic heat dissipation power. It can be concluded that the self-heating and HPM effects are different in the following two aspects: one is that the tendency of temperature variation caused by the two are different. The temperature variation under the influence of the self-heating effect shows a linear growth trend, while that under the influence of the HPM effect shows a stepwise rising sinusoidal signal-shaped trend. Due to the different operation modes of the two, the temperature rise rate and mechanism will be different. The gate voltage bias between the self-heating study (2 V, DC voltage) and the HPM study (300 V, 5 GHz sine wave) is disparate, resulting in a 10^8^ times difference in temperature rise rate. The HPM attack focuses on releasing high-power electromagnetic radiation within a nanosecond time range, causing the rapid heating and burning down of the equipment. The number of hot spots and distribution is the other difference. In analyzing the damage mechanism it was concluded that the left and right edge of the p-GaN region under the gate of the device is broken down under the action of HPM, and a large amount of breakdown current is generated, which causes the temperature to increase sharply and even burn out. The simulation results show that the two ends of the gate close to the source and drain with the action of HPM are potential damage areas, which are in good agreement with the experimental results. This is an important reference for research on HEMT damage location prediction technology and reliability enhancement.

## Figures and Tables

**Table 1:** The initial value (T = 300 K) of the key parameters.

Parameters	GaN	Al_0.23_GaN
Heat capacity *cv*	3.0 (J/(K·cm^3^))	2.76 (J/(K·cm^3^))
Thermal conductivity *κ*	1.3 (W/(K·cm))	1.66 (W/(K·cm))
Bandgap *Eg*_0_	3.51 (eV)	4.14 (eV)
Initial mobility *µ*_0_	1500 (cm^2^/(Vs))	1224 (cm^2^/(Vs))
Electron effective mass *m_e_*	0.22	0.24
Relative permittivity $\varepsilon_{r}$	9.4	9.26
